# Variation in Rotenone and Deguelin Contents among Strains across Four *Tephrosia* Species and Their Activities against Aphids and Whiteflies

**DOI:** 10.3390/toxins14050339

**Published:** 2022-05-13

**Authors:** Peiwen Zhang, Mengzi Zhang, Terri A. Mellich, Brian J. Pearson, Jianjun Chen, Zhixiang Zhang

**Affiliations:** 1 Key Laboratory of Natural Pesticide and Chemical Biology, Ministry of Education, South China Agricultural University, Guangzhou 510642, China; peiwen.zhang@ufl.edu; 2 Mid-Florida Research and Education Center, Department of Environmental Horticulture, Institute of Food and Agricultural Sciences, University of Florida, Apopka, FL 32703, USA; zhangmengzi@ufl.edu (M.Z.); tmellich@ufl.edu (T.A.M.); bpearson@ufl.edu (B.J.P.)

**Keywords:** *Aphis gossypii*, *Bemisia tabaci*, botanical pesticides, deguelin, rotenone, *Tephrosia adunca*, *Tephrosia candida*, *Tephrosia grandiflora*, *Tephrosia vogelii*

## Abstract

Botanical pesticides have received increasing attention for sustainable control of insect pests. Plants from the genus *Tephrosia* are known to produce rotenone and deguelin. Rotenone is known to possess insecticidal activities against a wide range of pests, but deguelin’s activities remain largely inconclusive. On the other hand, the biosynthesis of rotenone and deguelin may vary in *Tephrosia* species. This study analyzed the rotenone and deguelin contents in 13 strains across 4 *Tephrosia* species over 4 growing seasons using HPLC. Our study shows that the species and even the strains within a species vary substantially in the biosynthesis of rotenone and deguelin, and their contents can be affected by the growing season. After identification of the LC_50_ values of chemical rotenone and deguelin against *Aphis gossypii* (Glover) and *Bemisia tabaci* (Gennadius), leaf extracts derived from the 13 strains were used to test their insecticidal activities against the 2 pests. The results showed that the extracts derived from 2 strains of *T*. *vogelii* had the highest insecticidal activity, resulting in 100% mortality of *A. gossypii* and greater than 90% mortality of *B. tabaci*. The higher mortalities were closely associated with the higher contents of rotenone and deguelin in the two strains, indicating that deguelin also possesses insecticidal activities. This is the first documentation of leaf extracts derived from 13 *Tephrosia* strains against 2 important pests of *A. gossypii* and *B. tabaci*. The strain variation and seasonal influence on the rotenone and deguelin contents call for careful attention in selecting appropriate strains and seasons to produce leaf extracts for the control of insect pests.

## 1. Introduction

The genus *Tephrosia* is a member of the family Leguminosae and subfamily Papilionaceae, which is comprised of approximately 400 species that are widely distributed in tropical, subtropical, and arid regions of the world [1,2]. They are erect or prostrate herbs or soft or woody shrubs with dense branches and leaves, with heights ranging from 0.5 to 4 m [1]. One distinguished characteristic of *Tephrosia* species is their ability to biosynthesize phytochemical compounds with insecticidal activities. As a result, *Tephrosia* extracts have long been used as a botanical pesticide for control of a large number of insect pests [1,2].

Rotenone, a crystal with a chemical formula of C_23_H_22_O_6_ and molecular weight of 394.42, is a main insecticidal compound present in the leaves, flowers, roots, and seeds of *Tephrosia* plants [3]. Rotenone has been used to control leaf-feeding insects such as aphids and whiteflies in the order Hemipteran, caterpillars in the order Lepidoptera, as well as beetles, spiders, mites, carpenter ants, fleas, and lice on animals [4,5]. Rotenone is a selective and non-systemic pesticide with contact and stomach action. Its mode of action involves inhibition of the electron transport at the mitochondrial level, resulting in the blocking of phosphorylation from ADP to ATP, thus suppressing insect metabolism [1]. Synthesized rotenone is used legally in many countries, but it was banned by the United States (U.S.) Environmental Protection Agency (EPA) and the European Union (EU), and all non-piscicidal sales of rotenone were discontinued in Canada [6]. Policy changes in these countries and regions regarding the safety data for rotenone may have changed the commercial scope for botanical pesticides [7]. However, the use of plant extracts for insect control has been a tradition and remains strong throughout the rest of the world [1,7].

The species of *Tephrosia* vary significantly in the biosynthesis of rotenoid compounds. Sixteen species were evaluated by Irvine and Freyre [8], and 14 were found to biosynthesize some rotenoids. The highest amount of rotenoids was produced by *Tephrosia vogelii* Hook f., including rotenone, deguelin, rotenolone, and tephrosin in the leaves, petal, stems, and roots [1,9,10,11,12]. Leaves, however, accounted for 80–90% of the biosynthesized rotenoids [13]. Among the four rotenoid compounds, rotenone is reported to be the most toxic [1,2,3]. In addition, recent studies showed that the content of rotenoids differs among strains. Mkindi et al. [14] identified three chemotypes or strains within *T. vogelii* in East Africa. The dominant compounds in leaves of the chemotype 1 were deguelin and tephrosin, while the rotenone content was low. Chemotype 2, however, was rich in flavanones without the three rotenoids. Chemotype 3 contained all compounds identified in both chemotypes 1 and 2. These results suggest that the use of *Tephrosia* for the control of pests requires identification of the correct species and strains.

Here, we hypothesize that rotenoid content variation might not only be limited to the genetic resources in East Africa but also occur in those from other regions, and insecticidal activities may not only depend on rotenone but also other compounds, such as deguelin. Deguelin is a derivative of rotenone with a formula of C_23_H_22_O_6_ and molecular weight of 394.42 [15]. Deguelin has been found to have strong anticancer properties [10,16], but there is limited information about deguelin as a botanical pesticide [11,17], and no report is available about its mode of action as a botanical insecticide.

This study was intended to analyze the rotenone and deguelin contents in the leaves of 13 strains across 4 *Tephrosia* species during their growth over 4 seasons. The objectives were to determine if (1) variation in the rotenoid contents existed in the evaluated strains, (2) growing seasons affected the rotenoid contents, and (3) leaf extracts derived from the strains had insecticidal activities against *Aphis gossypii* (aphids) and *Bemisia tabaci* (whiteflies).

## 2. Results

### 2.1. Rotenoid Contents in 13 Strains

**Figure 1:**
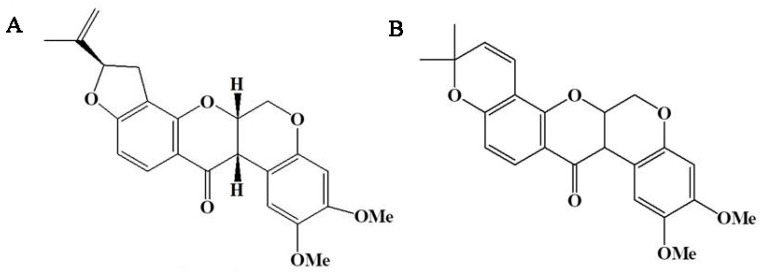
The structures of rotenone (**A**) and deguelin (**B**).

**Figure 2:**
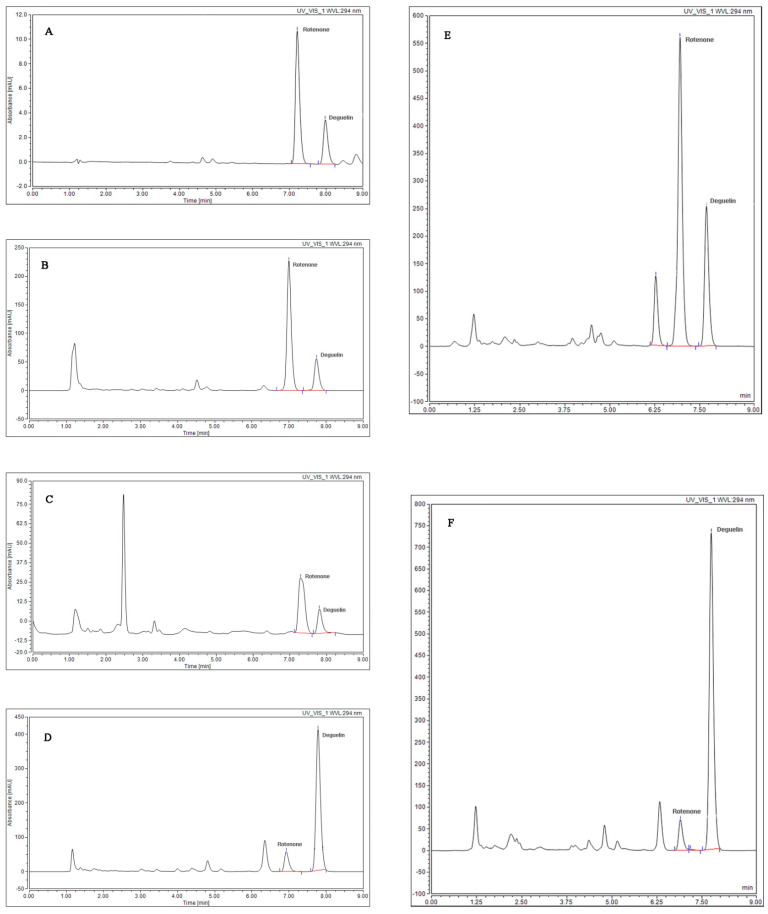
HPLC chromatographic profiles of rotenone and deguelin. (**A**) The chromatogram of standard rotenone and deguelin (25 μg/mL). (**B**–**D**) Rotenone and deguelin detected in the leaf extracts of *T. adunca*, *T. candida*, and *T. grandiflora*, respectively. (**E**,**F**) The chromatograms of rotenone and deguelin detected in leaf extracts of *T. vogelii* strains with purple flower (TVP) and white flower (TVW), respectively.

The structures of rotenone and deguelin are shown in Figure 1. The results of the HPLC analysis are presented in Figure 2. Rotenone and deguelin were detected 7.0 and 7.5 min following sample injection, respectively, and were identified by comparison to their retention times and ultraviolet (UV) spectra with reference standards. The leaf rotenone and deguelin contents in 13 strains across 4 species in November, February, May, and August are presented in Table 1 and Table 2.

The contents of rotenone in *T. adunca* derived from Venezuela (PI 308578 and PI 308579) ranged from 0.0215% to 0.2014% and 0.0195% to 0.0461%, respectively, across 4 seasons (Table 1). The rotenone contents in three *T.* *candida* strains collected from Brazil, South Africa, and the U.S. were lower than that of PI 308578 (*T. adunca* collected from Venezuela). *T. grandiflora* had the lowest rotenone content among the four species. *T. vogelii* collected from the U.S. (PI 257533) and Kenya (PI 305346) had the highest rotenone contents, being up to 0.8487% and 0.9713%, respectively. The rotenone contents in the other *T. vogelii* were much lower than those of PI 257533 and PI 305346 (Table 1).

The deguelin contents in the 2 *T. adunca* strains collected from Venezuela varied from 0.0211% to 0.1498% and 0.0211% to 0.2957%, respectively (Table 2). The contents of deguelin in *T. candida* derived from Brazil, South Africa, and the U.S. were 0.0000–0.0468%, 0.0000–0.0163%, and 0.0177–0.1444%, respectively. The deguelin contents in *T. grandiflora* collected from California ranged from 0.1065% to 2.1064% but differed from 0.0107% to 0.1412% in the strain collected from Arizona and from 0.0000% to 0.0424% in the strain derived from South Africa. The contents of deguelin in *T. vogelii* were generally higher than those the other species, of which the strains from Bolivia (PI 387870) and the U.S. (PI 574535) were the highest in February, being up to 4.0757% and 4.4269%, respectively.

Growing seasons affected the biosynthesis of rotenone and deguelin. Regardless of the strains, the rotenone contents were lower in November but generally higher from February to August (Table 1). On the other hand, the highest content of deguelin occurred in February, then decreased from May to August, and was lowest in November (Table 2).

### 2.2. Toxicity of Rotenone and Deguelin Chemicals against Aphids and Whiteflies

The toxic effects of rotenone and deguelin on aphids and whiteflies are tabulated in Table 3 and Table 4, respectively, which were based on the linear (y = m + bx) plots of the Probit curves for the two pests. Rotenone and deguelin are effectively toxic to aphids. The rotenone LC_50_ values at 24 and 48 h for the aphid nymphs were 2.38 and 2.06 mg/L, and they were 2.98 and 2.56 mg/L for adults, respectively (Table 3), while the 24- and 48-h LC_50_ values of deguelin for aphid nymphs were 9.03 and 7.75 mg/L and 12.10 and 10.88 mg/L for adults, respectively (Table 3). Compared with its effect on aphids, rotenone had lower toxicity to whiteflies, as the values at 24 and 48 h for LC_50_ were 14.84 and 12.41 mg/L for nymphs and 16.50 and 13.73 mg/L for adults, respectively (Table 4), but the 24- and 48-h LC_50_ values of deguelin for the nymphs were 53.95 and 37.94 mg/L, and they were 61.80 and 41.90 mg/L for adults, respectively. Thus, the toxicity of rotenone was stronger than deguelin for the two pests, and both rotenone and deguelin were more effective against aphids than whiteflies.

### 2.3. Toxicity of Tephrosia Leaf Extracts to Aphis gossypii and Bemisia tabaci

**Figure 3:**
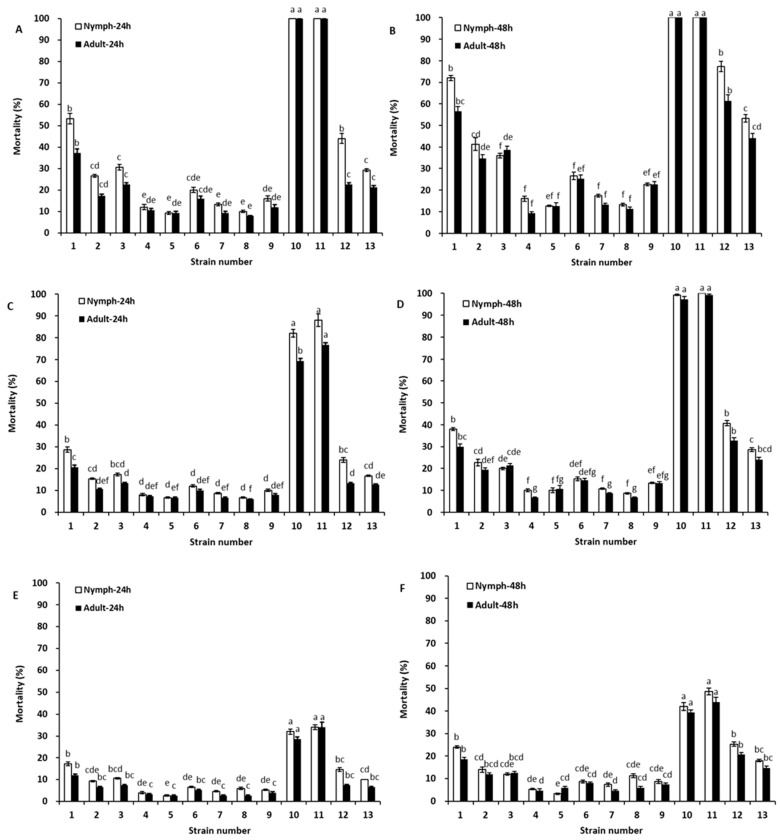
Insecticidal activity of leaf extracts derived from 13 strains of *Tephrosia* against nymph and adult aphids (*Aphis gossypii*). Aphid nymphs and adults were exposed to leaf extracts diluted with water at ratios of 1:500 (**A**,**B**), 1:1000 (**C**,**D**), and 1:1500 (**E**,**F**) by volume for 24 or 48 h, respectively. Error bars represent the standard error of the mean (*n* = 3). Statistical significance among samples was evaluated using one-way ANOVA followed by Tukey’s HSD test. Different letters upon the bars within the same period of insect exposure indicate significant difference at the *p* < 0.05 level. The numbers on the x-axes correspond the numbers of 13 strains of *Tephrosia* listed in Table 1 and Table 2.

The insecticidal activities of leaf extracts derived from 13 strains of *Tephrosia,* after being diluted to 1:500, 1:1000, and 1:1500 with water (*v*/*v*), were evaluated for their toxicity to nymph and adult aphids (Figure 3). Marked variation in the mortality of aphid nymphs and adults was observed among the *Tephrosia* strains and dilution rates (Figure 3). The mortality rates of aphids after 24 h of treatment with 1:500 diluted extracts derived from the 13 strains ranged from 8.00% to 100.00% and from 9.33% to 100.00% after 48 h of treatment. At the dilution ratio of 1:1000, the mortality percentages varied from 6.00% to 88.00% after 24 h of treatment as well as from 6.67% to 100.00% after 48 h of treatment. With the increased dilution, the mortality rates decreased, as the 1:1500 dilution resulted in mortality percentages that differed from 2.67% to 34.00% at 24 h as well as from 3.33% to 48.67% after 48 h of treatment. Regardless of the dilution, the insecticidal activities of the leaf extracts derived from *T. vogelii* collected from the U.S. (PI 257533) and Kenya (PI 305346) were the highest among the 13 strains due to the high contents of rotenone and deguelin (Table 1 and Table 2). The insecticidal activities of the extracts from *T. adunca* collected from Venezuela (PI 308578) and *T. vogelii* from Bolivia (PI 387870) and Puerto Rico (PI 574535) were significantly lower than those of PI 257533 and PI 305346, but the extracts derived from the remaining eight strains had even lower insecticidal activities.

**Figure 4:**
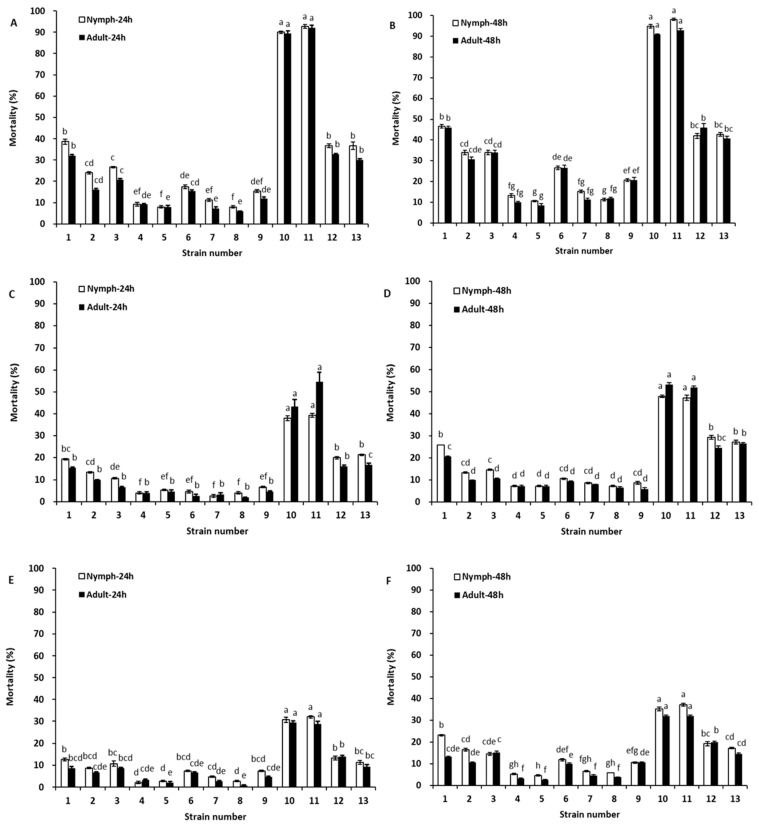
Insecticidal activity of leaf extracts derived from 13 strains of *Tephrosia* against nymph and adult *Bemisia tabaci*. Whitefly nymphs and adults were exposed to leaf extracts diluted with water at ratios of 1:100 (**A**,**B**), 1:300 (**C**,**D**), and 1:500 (**E**,**F**) by volume for 24 or 48 h, respectively. Error bars represent the standard error of the mean (*n* = 3). Statistical significance among samples was evaluated using one-way ANOVA followed by Tukey’s HSD test. Different letters upon the bars within the same period of insect exposure indicate significant difference at the *p* < 0.05 level. The numbers on the x-axes correspond the numbers of 13 strains of *Tephrosia* listed in Table 1 and Table 2.

The leaf extracts from the 13 strains were diluted to 1:100, 1:300, and 1:500 with water (*v*/*v*) and tested for control of *B. tabaci* nymphs and adults (Figure 4). At a leaf extract-to-water ratio of 1:100, the mortality percentages varied from 6.00% to 92.67% at 24 h and from 8.67% to 98.00% at 48 h. At a leaf extract dilution of 1:300, the mortality percentages ranged from 2.00% to 54.67% at 24 h and from 6.00% to 53.33% at 48 h. However, with a further dilution of 1:500, the mortality percentages differed from 0.67% to 32.00% at 24 h and from 2.67% to 37.33% at 48 h. For *B. tabaci*, the insecticidal activities of the strains from Puerto Rico (PI 257533) and Kenya (PI 305346) were the highest among the 13 strains due to high contents of rotenone and deguelin, followed by PI 308578, PI 387870, and PI 574535. The plant extracts from the other strains had lower insecticidal activities, which was similar to the results for aphids.

## 3. Discussion

There is increasing interest in botanical insecticides worldwide [9]. Botanical insecticides include dried, ground plant materials, crude plant extracts, or chemicals isolated from plants and used for pest management [1]. Plants from the genus *Tephrosia* are rich in phytochemicals, including rotenoids, and are used for control of a wide range of pests in Africa [14]. Recent reports show that species and even strains collected from East Africa vary in the content of rotenoids [12,14,18]. To achieve effective control of a pest, it is imperative to know exactly what species or strains should be used for pest management [1]. In the present study, we evaluated the rotenoid contents in 13 strains of *Tephrosia* across 4 species collected from a wide range of geographic areas and confirmed that the species and even strains within a species collected from areas other than East Africa vary in their rotenone and deguelin contents. Moreover, deguelin also possesses insecticidal activity against aphids and whiteflies.

Our results show substantial differences in the rotenone and deguelin contents among the evaluated strains within the same species and among different species (Table 1 and Table 2). The highest contents of rotenone and deguelin generally occurred in *T. vogelii*, while *T. grandiflora* had the lowest contents of the two chemicals. The levels of rotenone and deguelin in *T. adunca* and *T. candida* largely fell between those of *T. vogelii* and *T. grandiflora*. In addition to the difference among species, the rotenone and deguelin contents varied significantly among the strains within a given species. Furthermore, growth seasons also significantly affected the contents of the two compounds. The rotenone contents in strains of *T. vogelii* collected from Kenya (PI 305346), a country in East Africa, were the highest among the five *T. vogelii* strains in November and February, but their contents in May were comparable to and became lower than that of a strain collected from Puerto Rico (PI 257533) (Table 1). This result shows that the strains from places other than East Africa also contained comparably high levels of rotenone. In *T. adunca*, the content of rotenone in PI 308578 was 4–5 times greater than that of PI 308579. On average, deguelin was more abundant than rotenone. Similar to rotenone, *T. vogelii* had significantly higher levels of deguelin than the other species. The strains (PI 387870) collected from Bolivia had the highest deguelin content, followed by strains collected from Puerto Rico (PI 257533) and Kenya (PI 305346) (Table 2). These results again demonstrate that genetic resources derived from places other than East Africa contained higher rotenoid concentrations. The specific differences in both rotenone and deguelin are likely due to their genetic makeup. The differences among strains within a species were probably due to the adaptation to the environment, where soil nutrient and growth conditions may affect the biosynthesis of these compounds. Since the pathway for rotenone biosynthesis has not been completely established, biochemical analysis of strain differences remains challenging. One approach for gaining a better understanding of the differences is to use RNA-Seq technology to analyze transcript differences among the selected strains for identifying genes closely associated with variable differences in rotenone and deguelin contents. Nevertheless, our results confirm our hypothesis that variation in rotenone and deguelin occurs among *Tephrosia* species and even strains collected from regions other than East Africa. For effective control of pests, appropriate strains should be used.

In general, the highest concentrations of rotenone and deguelin occurred in February, and the lowest occurred in November. The seasonal variation in the two compound contents is probably related to the status of plant growth. The average temperature in winter in Apopka, Florida varies from 10 °C to 21 °C, and plant growth can either slow or stop. As a result, biosynthesis of secondary metabolites could be slow or decreased [19,20,21,22], resulting in lower contents of rotenone and deguelin in plants in November (Table 1 and Table 2). Seasonal variation in deguelin was also reported in *T. vogelii*, where higher concentrations occurred in the wet season compared with the dry season [17,23]. In the wet season, a significant variation in the highest and lowest levels of deguelin were observed in *T. vogelli* samples collected from the Same and Mbeya districts, based on the spatial and temporal variation [14]. Thus, in addition to choosing the correct strains of *T. vogelii*, understanding the seasonal variation in rotenone and deguelin contents in plants grown in a particular region is required for the use of leaf extracts to control the pests of interest.

To evaluate the insecticidal activities of leaf extracts, chemical rotenone and deguelin were first tested against *A. gossypii* and *B. tabaci*. Aphids and whiteflies are among the most destructive pests in the production of horticultural and field crops [24,25]. Evaluation of their responses may potentially lead to the direct application of the extracts for control of the two pests. Based on the LC_50_ values, rotenone and deguelin were active compounds against *A. gossypii* and *B. tabaci*. However, deguelin showed fewer toxic effects than rotenone. *A. gossypii* nymphs and adults were more susceptible to rotenone than deguelin, and the LC_50_ of value deguelin was about 3–4 times that of rotenone. This implies that if the rotenone is used in a greenhouse at the LC_50_ value, it will kill the aphids more easily than deguelin. Similarly, the LC_50_ value of deguelin was also about 3–5 times higher than that of rotenone for *B. tabaci*. Together, these indicate that rotenone is generally more effective than deguelin in controlling aphids and whiteflies. It is certain that rotenone and deguelin can enter aphids and whiteflies through their mouthparts, but it is unknown if they can enter the insects by penetrating the cuticle. Further studies are warranted to clarify this issue. Considering the fact that rotenone and deguelin have the same molecular formula and molecular weight, the difference in insecticidal activities may be attributed to their differences in configuration, which requires further investigation.

Our results demonstrate that the leaf extracts derived from different strains of *Tephrosia* plants differed in their effectiveness against aphids and whiteflies (Figure 3 and Figure 4). The high mortality was generally associated with strains with higher contents of rotenone and deguelin. The extracts derived from strains of *T. vogelii* collected from Puerto Rico (PI 257533) and Kenya (PI 305346) had higher rotenone and deguelin levels. Thus, higher mortality rates for aphids and whiteflies were observed after the application of their leaf extracts. These results agreed with reports by Mwangi [25] and Kayange et al. [26] that leaf extracts of *T. vogelii* and *T.* *candida* were effective against aphids and whiteflies. The effectiveness was traditionally attributed to the presence of rotenone, as it has been reported to be the most toxic among the rotenoids [15]. Limited attention has been given to deguelin. Deguelin, however, is the most abundant rotenoid in *T. vogelii* extracts [26,27], and the same was true in this study (Table 1 and Table 2). Although the insecticidal activity of rotenone is 3–5 times higher than that of deguelin (Table 3 and Table 4), when the extract of PI 257533 and PI 305346 of *T. vogelii* is applied, the high content of deguelin may be more than enough to make up for its low activity (Figure 3 and Figure 4). Belmain et al. [17] reported that rotenone itself played a relatively minor role in the control of cowpea weevils (*Callosobruchus maculatus*) compared with the more abundant compound deguelin. Deguelin was most effective against adult bruchids when 2% leaf extract of *T. vogelii* was applied to cowpeas [28]. Our study shows that leaf extracts derived from two strains of *T. vogelii* (PI 257533 and PI 305346) can effectively control *A. gossypii* and *B. tabaci*.

The results from this study provide much-needed information on the sustainable use of *Tephrosia* in the control of insect pests. First, *T. vogelii* should be considered an appropriate choice of species. Second, strains of *T. vogelii* should be analyzed seasonally to determine which strains in which seasons produce higher concentrations of rotenone and deguelin. Third, leaves should be collected from the designated strains at the particular seasons and used for extraction. Fourth, appropriate dilutions should be tested for effectiveness in the control of particular insect pests. Additionally, caution should be given for maintaining insecticidal activities, as rotenoids can quickly degrade when exposed to direct sunlight and high temperatures. Moreover, insecticidal efficacy could be improved by simply incorporating detergents such as liquid soap [17].

## 4. Conclusions

This study analyzed the rotenone and deguelin contents of 13 strains across 4 species (*T. adunca*, *T. candida*, *T. grandiflora*, and *T. vogelii*) collected mainly from North and South America and documented that the species and strains varied greatly in biosynthesis of the two compounds. Some strains were able to produce rotenone and deguelin at concentrations that were either comparable to or higher than a strain collected from Kenya, suggesting significant genetic variation in biosynthesis of the two compounds occurs in strains other than those collected from East Africa. Additionally, the biosynthesis of rotenone and deguelin varied according to the season. Leaf extracts derived from two strains (PI 257533 and PI 305346) of *T. vogelii* can effectively control *A. gossypii* and *B. tabaci*. As there is increasing interest in botanical pesticides, the findings from this study provide concrete evidence to show that selected strains of *T. vogelii* could be valuable genetic resources for producing extracts to control important pests such as aphids and whiteflies in a sustainable manner.

## 5. Materials and Methods

### 5.1. Plant Materials

Seeds of 12 strains across *T. adunca*, *T. candida*, *T. grandiflora*, and *T. vogelii* were provided by the U.S. National Plant Germplasm System (NPGS) in Griffin, Georgia and the Desert Legume Program (DELEP) at the University of Arizona in Tucson, AZ, U.S. One strain of *T. candida* was collected locally in Apopka, FL, U.S. The seeds were sown in a substrate composed of 60% peat, 20% perlite, and 20% sand by volume, and the seedlings were grown in 3-gallon (11.4 L) plastic containers surface-applied with a controlled-release fertilizer—Osmocote Plus 8–9 month 15-9-12 (ICL Specialty Fertilizers, Dublin, OH, USA)—at 20 g per pot. After 1 year of growth, the plant morphologies of the healthy and uniform varieties were recorded. More than 10 plants per variety were used for the experiments. Fresh leaves from different plants were collected in 2020–2021 in different seasons in the city of Apopka, Florida, U.S. at an altitude above sea level of 131 feet (28°40′34″ N–81°30′43″ W). The collected leaves were dried at 60 °C in a heat chamber for 48 h. The dried plant materials were then crushed into powders and kept separately in airtight plastic bags in the dark.

### 5.2. Sample Preparation for Evaluation of Rotenone and Deguelin Contents in Acetonitrile Extracts

A precisely weighed amount (1.0 g) of the powdered plant material was extracted with 15 mL of acetonitrile by ultrasonic treatment for 2 h and then placed in a 4 °C refrigerator, with ultrasonic extraction occurring for 2 h every 12 h in a cold-soaking extraction solution for 3 days. The extract was filtered into a centrifuge tube with the addition of 1 g anhydrous sodium sulfate and 1 g sodium chloride for the cleanup. The mixture was then shaken vigorously for 1 min and stranded for stratification. An aliquot from the upper layer was evaporated to near dryness with a vacufuge concentrator (Eppendorf, Hamburg, Germany) at 30 °C. The residual extract was dissolved in acetonitrile, filtered through a 0.2-μm filter, and placed in a 2-mL vial for HPLC analysis. The extracts were kept in a refrigerator at 4 °C and in the dark until analysis could be completed. An HPLC system (UltiMate 3000 HPLC, Thermo Fisher Scientific Inc., Waltham, MA, USA) with an ultraviolet visible detector was used for the rotenone and deguelin concentration analysis. Chromatography was performed using an Accucore™ C_18_ HPLC column (reversed phase, 150 mm × 3 mm, 2.6 μm particle size, Thermo Scientific) with acetonitrile/water (50/50, *v*/*v*) as the mobile phase solution. The injection volume was 1 μL, and separation was carried out at 25 °C with a flow rate of 0.5 mL/min. The detection wavelength was set to 294 nm and the total run time to 9 min. The experiment was designed with three replications.

### 5.3. Standard Calibration

A rotenone standard and deguelin standard were purchased from Thermo Fisher Scientific Inc. (Waltham, MA, USA). The solid rotenone standard and deguelin standard were dissolved in 100% methanol at a rate of 25 µg/mL. Standard calibration was achieved by performing serial injection volumes at rates of 0.2, 1, 5, 8 and 10 µL, resulting in a calibration correlation of R^2^ = 0.99.

### 5.4. Toxicity of Rotenone, Deguelin, and Leaf Extracts of Tephroisa Plants against Aphis gossypii

Adult *Aphis gossypii* (Glover) were collected from the University of Florida’s Mid-Florida Research and Education Center (MREC) in Apopka, FL, U.S. The stock colony of aphids was maintained on hibiscus plants in entomological cages (91.4 × 61 × 61 cm). Entomological cages were kept in an acclimatization building at 25 °C, 65 ± 5% relative humidity (RH), and a 14:10-h light:dark (L:D) photoperiod.

Double-sided tape was stuck on a glass slide. Aphids at the fourth nymph or the emergence aphids (nymph and adult, respectively) were picked up with a small brush, and 50 aphids were placed on the double-sided tape. Rotenone and deguelin were diluted with water into 5 concentrations (0.5, 1, 2, 4, and 8 µg/mL of rotenone and 1, 2, 4, 8, and 16 µg/mL of deguelin, respectively), and the above-mentioned aphid glass slide was immersed in the solutions for about 15 s. After the excess solution was absorbed with absorbent paper, the glass slide was placed into a 9-cm in diameter Petri dish with a moistened paper. All Petri dishes were placed in a room with a constant temperature of 25 °C, L:D = 14:10, and an RH of 70%. After 24 and 48 h, the mortality rates of the aphids were checked under a dissecting microscope. Aphids that did not move after a gentle touch with the tip of a brush were deemed to be dead. There were three replicates per treatment, and the control was the glass slides soaked with water. The virulence regression equation, the lethal concentration (LC_50_) of rotenone and deguelin, and the 95% confidence limit of the LC_50_ value were calculated by the least squares method. Subsequently, the samples of dry leaves for each strain were extracted according to the extraction method mentioned above and diluted 500, 1000, and 1500 times with water by volume for evaluating their insecticidal activities against aphids. The biological activities of the extracts were evaluated in the same way as the above-mentioned rotenone and deguelin after 24 and 48 h of exposure, and the aphid mortalities were recorded.

### 5.5. Toxicity of Rotenone, Deguelin, and Leaf Extracts of Tephroisa Plants against Bemisia tabaci

Adult *Bemisia tabaci* (Gennadius) were collected from lima beans (*Phaseolus lunatus*) at the MREC in Apopka, FL, U.S. The stock colony of whiteflies was maintained on lima bean plants in entomological cages (91.4 × 61 × 61 cm) constructed with aluminum and anti-aphid mesh. The entomological cages were kept in an acclimatization building at 25 °C, 65 ± 5% RH, and a 14:10-h L:D photoperiod.

Rotenone and deguelin were diluted with water into 5 concentrations (3.125, 6.25, 12.5, 25, and 50 µg/mL of rotenone and 10, 20, 40, 80, and 160 µg/mL of deguelin), and the leaf extracts were diluted 100, 300, and 500 times with water by volume. The leaves of the lima beans were evenly sprayed with the above-mentioned solutions until runoff. The control plant was sprayed with water (containing the same concentration of organic solvents). After the leaves dried, the treated plants and the control plants were placed in an insect cage, and 50 nymph and adult whiteflies were released into the cage. The survival of the whiteflies was checked after 24 h and 48 h. Whiteflies that did not move after a gentle touch with the tip of a brush were regarded as dead. The experiment was set up as a randomized complete block design with 3 replications. The control group was water. The virulence regression equation, the lethal concentration (LC_50_) of rotenone and deguelin on whiteflies, and the 95% confidence limit of the LC_50_ value were calculated by the least squares method. The mortality rates of the whitefly nymphs and adults were recorded.

### 5.6. Statistical Analysis

The rotenone and deguelin contents in 13 strains were subjected to analysis of variance (ANOVA) using SPSS software (version 19.0, IBM Corp., Armonk, NY, USA), and the mean significance across strains and the strains within a species were separated by Tukey’s Honestly Significant Difference (HSD) test at the *p* < 0.05 level. The virulence regression equation, the lethal concentration (LC_50_) values with their corresponding 95% confidence limits, and the correlation coefficients were calculated using Probit analysis and DPS software. All tests in this study were performed in triplicate. The data are shown as the mean ± SE.

## Figures and Tables

**Table 1:** Rotenone contents in leaves of 13 strains of *Tephrosia* across 4 species collected from different regions.

NO.	Species	Origin	Content (% in m/m)			
			November	February	May	August
1	*Tephrosia adunca*	Venezuela (PI 308578)	0.0215 ± 0.0003 aDE ^z^	0.2014 ± 0.0163 aC	0.1502 ± 0.0179 aB	0.1375 ± 0.0004 aC
2	*Tephrosia adunca*	Venezuela (PI 308579)	0.0195 ± 0.0009 aE	0.0461 ± 0.0080 bDE	0.0356 ± 0.0035 bC	0.0293 ± 0.0002 bJ
3	*Tephrosia candida*	Brazil (PI 337100)	0.0257 ± 0.0001 bC	0.1741 ± 0.0045 aCD	0.1198 ± 0.0538 aB	0.1012 ± 0.0043 aE
4	*Tephrosia candida*	South Africa (DLEG 990070)	0.0422 ± 0.0008 aA	0.1198 ± 0.0929 aCDE	0.1172 ± 0.0073 aB	0.0821 ± 0.0009 bF
5	*Tephrosia candida*	US, Florida (PI No. unknown)	0.0089 ± 0.0004 cF	0.1984 ± 0.0120 aC	0.1202 ± 0.0066 aB	0.0663 ± 0.0001 cG
6	*Tephrosia grandiflora*	US, California (DLEG 900611)	0.0000 ± 0.0000 aH	0.0188 ± 0.0101 aE	0.0342 ± 0.0019 aC	0.0370 ± 0.0004 aI
7	*Tephrosia grandiflora*	US, Arizona (DLEG 930029)	0.0000 ± 0.0000 aH	0.0043 ± 0.0022 aE	0.0058 ± 0.0001 bC	0.0012 ± 0.0001 bK
8	*Tephrosia grandiflora*	South Africa (DLEG 910177D)	0.0000 ± 0.0000 aH	0.0022 ± 0.0011 aE	0.0062 ± 0.0001 bC	0.0013 ± 0.0001 bK
9	*Tephrosia vogelii*	Congo (PI 213396)	0.0030 ± 0.0002 dG	0.0242 ± 0.0054 cE	0.0264 ± 0.0011 cC	0.0494 ± 0.0001 eH
10	*Tephrosia vogelii*	US, Puerto Rico (PI 257533)	0.0229 ± 0.0012 bD	0.7498 ± 0.0594 bB	0.6658 ± 0.0455 aA	0.8487 ± 0.0041 aA
11	*Tephrosia vogelii*	Kenya (PI 305346)	0.0389 ± 0.0023 aB	0.9713 ± 0.1060 aA	0.7034 ± 0.0316 aA	0.8110 ± 0.0007 bB
12	*Tephrosia vogelii*	Bolivia (PI 387870)	0.0221 ± 0.0006 bD	0.0141 ± 0.0089 cE	0.1524 ± 0.0334 bB	0.0847 ± 0.0037 dF
13	*Tephrosia vogelii*	US, Puerto Rico (PI 574535)	0.0112 ± 0.0005 cF	0.0108 ± 0.0071 cE	0.0743 ± 0.0152 bcBC	0.1285 ± 0.0017 cD

^z^ Statistical significance among samples was evaluated using one-way ANOVA followed by Tukey’s HSD test. Values represent mean ± standard error (*n* = 3). Different lowercase letters after the means within a column indicate significant difference within the same species, and different uppercase letters after the means indicate significant difference across species at the *p* < 0.05 level.

**Table 2:** Deguelin contents in leaves of 13 strains of *Tephrosia* across 4 species collected from different regions.

NO.	Species	Origin	Content (% in m/m)			
			November	February	May	August
1	*Tephrosia adunca*	Venezuela (PI 308578)	0.0211 ± 0.0006 aE ^z^	0.1498 ± 0.0192 aC	0.0901 ± 0.0084 bD	0.1101 ± 0.0003 bF
2	*Tephrosia adunca*	Venezuela (PI 308579)	0.0211 ± 0.0012 aE	0.2230 ± 0.0149 aC	0.1485 ± 0.0106 aD	0.2957 ± 0.0012 aE
3	*Tephrosia candida*	Brazil (PI 337100)	0.0000 ± 0.0000 bF	0.0468 ± 0.0020 aC	0.0158 ± 0.0024 bD	0.0297 ± 0.0008 bH
4	*Tephrosia candida*	South Africa (DLEG 990070)	0.0000 ± 0.0000 bF	0.0163 ± 0.0082 aC	0.0140 ± 0.0013 bD	0.0045 ± 0.0045 cI
5	*Tephrosia candida*	US, Florida (PI No. unknown)	0.0177 ± 0.0010 aE	0.1444 ± 0.0662 aC	0.1101 ± 0.0019 aD	0.0777 ± 0.0090 aG
6	*Tephrosia grandiflora*	US, California (DLEG 900611)	0.1062 ± 0.0021 aCD	2.1064 ± 0.3909 aB	1.1226 ± 0.0774 aC	0.7939 ± 0.0157 aC
7	*Tephrosia grandiflora*	US, Arizona (DLEG 930029)	0.0111 ± 0.0008 bEF	0.1412 ± 0.0568 bC	0.0107 ± 0.0011 bD	0.0118 ± 0.0002 bHI
8	*Tephrosia grandiflora*	South Africa (DLEG 910177D)	0.0000 ± 0.0000 cF	0.0424 ± 0.0025 bC	0.0218 ± 0.0037 bD	0.0125 ± 0.0001 bHI
9	*Tephrosia vogelii*	Congo (PI 213396)	0.0949 ± 0.0028 cD	1.8263 ± 0.3812 cB	0.9442 ± 0.0515 cC	0.7809 ± 0.0086 cC
10	*Tephrosia vogelii*	US, Puerto Rico (PI 257533)	0.1046 ± 0.0092 cCD	2.5960 ± 0.2347 bcB	1.0358 ± 0.2452 cC	0.4178 ± 0.0004 dD
11	*Tephrosia vogelii*	Kenya (PI 305346)	0.1129 ± 0.0023 cC	2.4223 ± 0.2885 cB	1.1009 ± 0.1177 cC	0.4389 ± 0.0010 dD
12	*Tephrosia vogelii*	Bolivia (PI 387870)	0.6222 ± 0.0107 aA	4.0757 ± 0.1076 abA	2.0828 ± 0.2709 bB	1.3542 ± 0.0110 bB
13	*Tephrosia vogelii*	US, Puerto Rico (PI 574535)	0.3102 ± 0.0102 bB	4.4269 ± 0.9611 aA	2.9027 ± 0.1128 aA	2.0860 ± 0.0168 aA

^z^ Statistical significance among samples was evaluated using one-way ANOVA followed by Tukey’s HSD test. Values represent mean ± standard error (*n* = 3). Different lowercase letters after the means within a column indicate significant difference within the same species, and different uppercase letters after the means indicate significant difference across species at the *p* < 0.05 level.

**Table 3:** Toxicities of rotenone and deguelin against nymph and adult aphids (*Aphis gossypii*).

Treatment	Target Organism	Time	LC_50_ (mg/L)	95% Confidence Limit (mg/L)	Regression Equations (Y = m + bx)	R^2^
Rotenone	Nymph	24 h	2.38	2.02–2.81	Y = 3.9472 + 2.7919x	0.9543
		48 h	2.06	1.74–2.43	Y = 4.1754 + 2.6329x	0.9428
	Adult	24 h	2.98	2.46–3.60	Y = 3.7602 + 2.6177x	0.9775
		48 h	2.56	2.12–3.09	Y = 4.0216 + 2.3956x	0.9532
Deguelin	Nymph	24 h	9.03	7.10–11.48	Y = 2.5669 + 2.5462x	0.9966
		48 h	7.75	5.99–10.03	Y = 3.1992 + 2.0250x	0.9880
	Adult	24 h	12.10	8.74–16.75	Y = 2.6502 + 2.1701x	0.9738
		48 h	10.88	7.43–15.91	Y = 3.3679 + 1.5746x	0.9579

**Table 4:** Toxicities of rotenone and deguelin against nymph and adult whiteflies (*Bemisia tabaci*).

Treatment	Target Organism	Time	LC_50_ (mg/L)	95% Confidence Limit (mg/L)	Regression Equations (Y = m + bx)	R^2^
Rotenone	Nymph	24 h	14.84	11.84–18.61	Y = 2.9108 + 1.7835x	0.9862
		48 h	12.41	9.97–15.44	Y = 3.0114 + 1.8184x	0.9733
	Adult	24 h	16.50	13.10–20.79	Y = 2.8053 + 1.8027x	0.9866
		48 h	13.73	10.85–17.38	Y = 3.0999 + 1.6702x	0.9740
Deguelin	Nymph	24 h	53.95	42.74–68.09	Y = 1.8779 + 1.8027x	0.9942
		48 h	37.94	30.79–46.74	Y = 1.9612 + 1.9245x	0.9868
	Adult	24 h	61.80	49.07–77.82	Y = 1.4796 + 1.9656x	0.9860
		48 h	41.90	33.79–51.95	Y = 1.9916 + 1.8545x	0.9912

## Data Availability

Data are contained within the article.
